# Eukaryotic Recombinases Duplicated After Divergence From Known Asgard Archaeal RadA: Implications for the Evolution of Sex During Eukaryogenesis

**DOI:** 10.1093/gbe/evaf240

**Published:** 2025-12-23

**Authors:** Lisa Matsuo, Anna M G Novák Vanclová, Andrew Pomiankowski, Nick Lane, Joel B Dacks

**Affiliations:** Centre for Life's Origins and Evolution, Department of Genetics, Evolution and Environment, University College London, London WC1E 6BT, UK; Division of Infectious Diseases, Department of Medicine, and Department of Biological Sciences, University of Alberta, 1-124 Clinical Sciences Building, 11350-83 Avenue, Edmonton T6G 2G3, Canada; Centre for Life's Origins and Evolution, Department of Genetics, Evolution and Environment, University College London, London WC1E 6BT, UK; Centre for Life's Origins and Evolution, Department of Genetics, Evolution and Environment, University College London, London WC1E 6BT, UK; Centre for Life's Origins and Evolution, Department of Genetics, Evolution and Environment, University College London, London WC1E 6BT, UK; Division of Infectious Diseases, Department of Medicine, and Department of Biological Sciences, University of Alberta, 1-124 Clinical Sciences Building, 11350-83 Avenue, Edmonton T6G 2G3, Canada; Institute of Parasitology, Biology Centre, Czech Academy of Sciences, Ceské Budějovice (Budweis) 370 05, Czech Republic

**Keywords:** Rab51, DMC1, evolutionary cell biology, LECA, meiosis

## Abstract

The origin of meiotic sex was a key milestone in the evolution of the eukaryotic cell. The paralogous DNA recombinases Rad51 and meiosis-specific DMC1 are nearly universal among eukaryotes and have been used previously to trace the timing and origins of the meiotic machinery. Here we perform comparative genomics and phylogenetic analyses of Rad51 and DMC1 drawn from diverse eukaryotes with RadA recombinase sequences from a broad sampling of archaeal taxa, focusing on the recently sequenced diversity of Asgard archaeal taxa. We show that even with increased and new sampling, the eukaryotic Rad51 and DMC1 proteins still resolve separately from any archaeal RadA sequences. These findings suggest that the duplication of RadA into general and meiosis-specific paralogues occurred after the divergence of the eukaryotic progenitor and did not evolve at an earlier stage. These findings raise the important question of how the evolution of meiotic sex was linked to genome size expansion and the acquisition of the mitochondrial endosymbiont in early eukaryotes.

Significance Understanding the series of events and selective pressures that drove the evolution of eukaryotes is one of the major mysteries in evolutionary biology, with the origin of sex being a key point to resolve. Past work showed that the eukaryotic recombinases Rad51 and DMC1 have archaeal origins, but with the explosion of newly sampled archaeal genome sequence data, the timing of their origins from archaeal ancestors deserves revisiting. Here we show that even with this new sampling, the emergence of the meiotic-specific DMC1 paralogue occurred after the divergence of eukaryotes from sampled archaea. We integrate implications of this timing, with published data from other eukaryotic systems (e.g. endomembrane, mitochondria, cytoskeleton) to suggest a possible ordering of some evolutionary innovations during eukaryogenesis.

## Introduction

The evolutionary transition from prokaryotic to eukaryotic cellular organization, termed eukaryogenesis (Donoghue et al. 2023; Vosseberg et al. 2024), was arguably the most fundamental shift in cellular history. It is also one of the more highly contested areas of study in evolutionary biology (O’Malley 2010). Across the span of theories for eukaryogenesis (Martin and Müller 1998; Cavalier-Smith 2002; Devos 2021; Guglielmini et al. 2022; López-García and Moreira 2023), there is consensus that the process involved the symbiosis of prokaryotic lineages, each one contributing genetic and cellular machinery that it had independently developed, to the new eukaryotic configuration. While the possibility of additional players cannot be ruled out, it is generally agreed that there were two main prokaryotic contributors (Donoghue et al. 2023; Vosseberg et al. 2024). An alpha proteobacterial lineage gave rise to the mitochondrion, albeit the precise identity of that lineage remains an interesting subject of inquiry (Muñoz-Gómez et al. 2022). This became an endosymbiotic partner within a lineage of Asgardarchaeota, the latter contributing the cytoskeleton, several endomembrane system components, and nuclear house-keeping machinery (Spang et al. 2015; Akıl and Robinson 2018; Eme et al. 2023; Wollweber et al. 2025). The resulting eukaryotic organism was a cellular and genomic chimera.

Determining the order in which eukaryotic traits such as the nucleus, mitochondria, endomembrane systems, and cytoskeleton evolved during eukaryogenesis is crucial to understanding the events and forces that initiated the evolution of, and culminated in, the population of organisms that gave rise to all living eukaryotes (Richards et al. 2024), i.e. the Last Eukaryotic Common Ancestor (LECA). One concrete way of addressing eukaryogenesis is to trace the prokaryotic origins of key cellular machinery and assess whether the eukaryotic version of each machinery is present in modern prokaryotic lineages, and by implication evolved before, during or after the establishment of the endosymbiotic partnership (Richards et al. 2024).

The capacity for sexual reproduction is nearly ubiquitous across eukaryotic life, with a deeply conserved dance of cell fusion and a two-step meiosis that involves reciprocal exchange across the whole nuclear genome (Colnaghi et al. 2020; Bergero et al. 2021). A two-step meiosis was inferred to be present in the LECA (Dacks and Roger 1999; Ramesh et al. 2005; Schurko et al. 2009) and has long been recognized as a critical step in eukaryogenesis (Bell 1982). The advent of sexual reproduction would have facilitated the evolution of large genomes, compared with organisms that rely on lateral gene transfer (LGT) (Koonin et al. 2004; Makarova et al. 2005; Colnaghi et al. 2020, 2022). In particular, unlike LGT, reciprocal recombination and homologous pairing along chromosomes in meiotic sex enabled the maintenance of far larger numbers of protein-coding genes in the face of deleterious mutation pressure (Colnaghi et al. 2020). It also permitted the expansion of repeat sequences such as gene families and selfish genetic elements, which are characteristic features of eukaryotic genomes (Hickey 1993). Notably, the symbiotic partner that became mitochondria did not evolve meiotic recombination, is largely uniparentally inherited and as a consequence is genetically depauperate in the extreme (Radzvilavicius et al. 2017).

One of the most important prokaryotic enzymes involved in genetic recombination is the RecA gene. The RecA protein is essential for DNA repair, homologous recombination and genome stability. RecA has both bacterial and archaeal (RadA) homologs and two paralogues in eukaryotes (Rad51 and DMC1) (Seitz et al. 1998). Rad51 is expressed in both meiotic and mitotic cells and plays a key role in double-strand break repair, whereas DMC1 is expressed solely during meiosis and actively promotes strand exchange in homologous recombination (Brown and Bishop 2014). Rad51 and DMC1 are inferred to have archaeal ancestry, being derived from RadA (Seitz et al. 1998), though past analyses have not found any archaea that encode the duplicated paralogues corresponding to Rad51 and DMC1. But there has been a tremendous recent growth in the discovery of archaea, especially Asgard archaea (eg. Eme et al. (2023)), as well as a large expansion of eukaryotic representation (see Leka & Wideman (2024)). Therefore, in order to better understand the timing of the duplication that gave rise to the meiosis-specific eukaryotic homologs, we undertook a molecular evolutionary analysis of RadA, Rad51, and DMC1 using updated and extended genomic sampling.

## Results

In order to address the question of when the recombinases evolved during eukaryogenesis, a comprehensive search was first conducted for Rad51 and DMC1 proteins across a deep and balanced sampling of eukaryotes. Starting from the curated set of eukaryotic genomic and transcriptomic datasets in the EukProt repository (Leka and Wideman 2024), homology searching and validation of ortholog assignment by phylogenetics was used to identify paralogues of both Rad51 and DMC1 in this taxonomically representative sampling of known eukaryotic diversity (Fig. S1).

Our phylogenetic analysis reconstructed well-supported clades of Rad51 vs DMC1, each containing a broad diversity of eukaryotes. Notably, both clades contained representatives from the major eukaryotic supergroups that are proposed to span the eukaryotic root (Williamson et al. 2025) including representatives from the Discoba and from the Metamonada (e.g. *Trichomonas vaginalis* that encodes both Rad51 and DMC1). These data also included sampling from the enigmatic sister-taxon to parabasalids *Anaeramoeba ignava*, the genome of which was recently reported (Jerlström-Hultqvist et al. 2024). Consistent with other reports of unusual meiotic machinery in that taxon (Gallot-Lavallée et al. 2023), we detected no DMC1, but Rad51 was found. Understanding the sexual cycle of *Anaeramoeba* will be particularly fascinating, given its need to ensure vertical inheritance of the unusual symbiotic organelle (i.e. symbiosome) that it contains (Jerlström-Hultqvist et al. 2024). Another notable observation was the DMC1 protein identified in the recently described anaerobic flagellate *Barthelona* sp. PAP020 (Yazaki et al. 2020), from the BaSk clade related to the prominent metamonad lineage Fornicata. While the presence of a DMC1 paralog is not entirely unexpected, this is an important data point as it is the first molecular evidence in support of a sexual cycle for this protist, a life stage that has not yet been microscopically observed. Thus, we are confident that our sampling is consistent with both Rad51 and DMC1 proteins being present in the LECA population, including the new phylogenetic definition (Williamson et al. 2025) and updated eukaryotic genomic sampling.

The Rad51 and DMC1 homologs identified were then used to construct HMM profiles which were used as queries for searches into a custom dataset of genomes and Metagenomically Assembled Genomes (MAGs) of archaea (Table S1). This yielded a preliminary dataset, which was merged with the eukaryotic set. Phylogenetic analysis of this larger dataset allowed us to identify identical, highly divergent, or RadB sequences which had erroneously been drawn in by the sensitive HMM searches (Fig. S2).

**Fig. 1.**
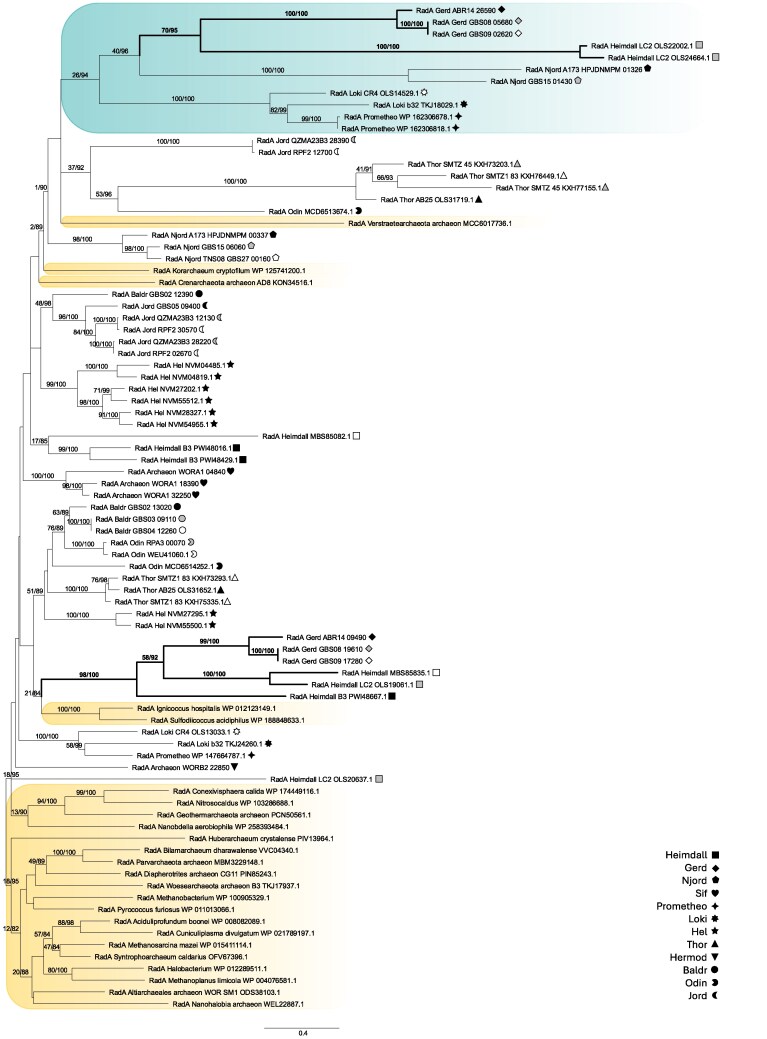
Two RadA clades in a subset of Asgard archaea. This phylogenetic analysis of selected Asgard archaeal RadA homologs reconstructs a moderately supported clade of RadA paralogues (green shade) encompassing Heimdallarchaea, Njordarchaea, Gerdarchaea, and less robustly Lokiarchaea, distinct from paralogues from the same MAGs in the analysis. Highly supported subclades representing these paralogs in Heimdallarchaea and Gerdarchaea are highlighted with higher line weight. Asgard archaeal taxa are denoted by different symbols, as per inset legends, while the symbol shading denotes proteins from the same MAG taxon. Yellow shading denotes RadA homologs from other groups of archaea which did not form as a well-supported monophylum in this analysis. Support values for 100 NP bootstraps and % of 1000 UF bootstraps for IQ-TREE2 analyses are shown at each node supported by greater than 50 NP or 80 UF.

Using only the archaeal dataset, two clear, moderately supported clades of Asgard archaeal sequences were found (Fig. 1), suggesting a duplication of RadA, at the very least in the subgroup uniting Heimdallarchaea and Gerdarchaea, given the relatively robust support for those clades (70/95 and 98/100, respectively, both clades are highlighted with higher line weights).

**Fig. 2.**
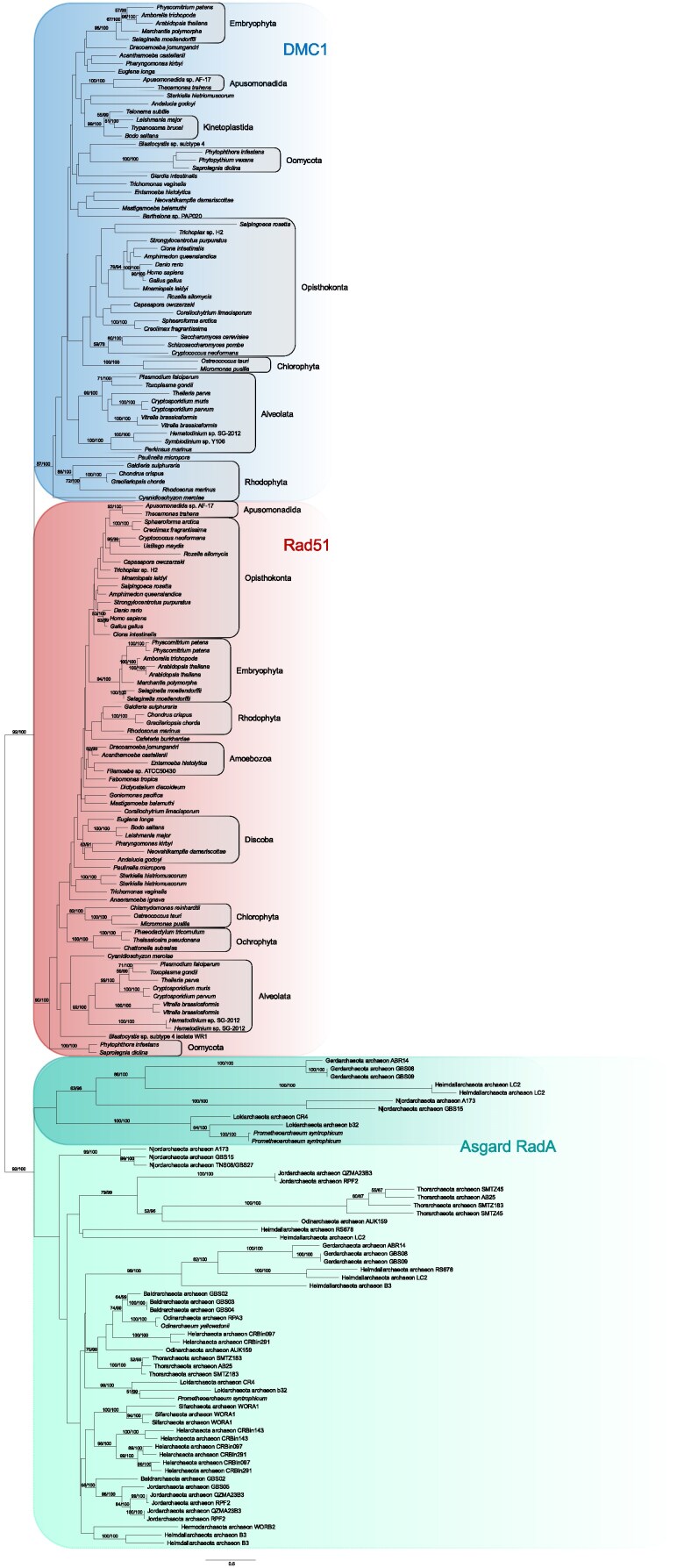
Rad51 and DMC1 form robust eukaryote-specific clades in analysis with all sampled Asgard archaeal RadA paralogues. Support values for 100 NP bootstraps and % of 1000 UF bootstraps for IQ-TREE2 analyses are shown at each node supported by greater than 50 NP.

**Fig. 3.**
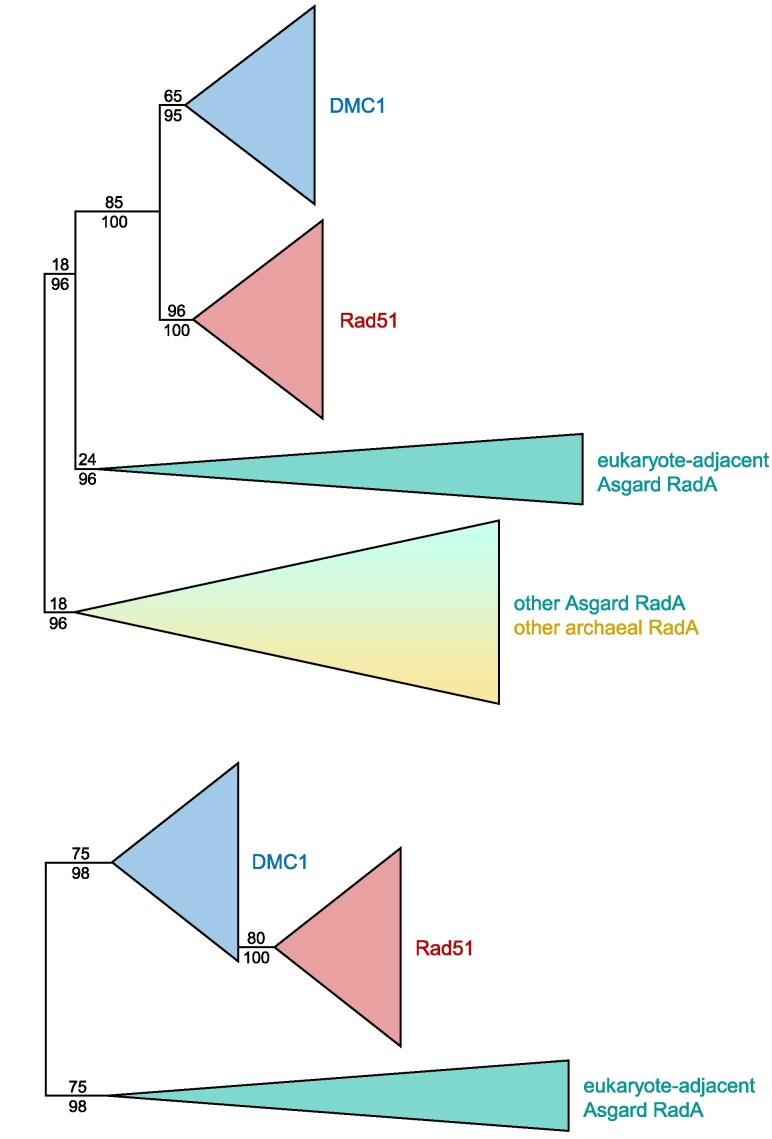
Rad51 and DMC1 form robust eukaryote-specific clades in analyses with different selections of outgroup taxa. These schematics illustrate the results analyzing the pan-eukaryotic dataset outgroup rooted by a) the set of RadA paralogues that include Asgard archaeal and non-Asgard archaeal taxa (data shown in full in Fig. S3) and b) the set of Asgard archaeal RadA sequences that grouped most closely to eukaryotic paralogs in Fig. S3 (data shown in full in Fig. S4). Support values for 100 NP bootstraps and % of 1000 UF bootstraps for IQ-TREE2 analyses are shown only for the clade-defining nodes of interest.

Finally, assessment was made of the placement of the eukaryotic recombinases within the prokaryotic RadA homologs. Regardless of whether the analyzed datasets included Asgard archaeal and eukaryotic homologs only (Fig. 2), non-Asgard archaeal outgroups (Fig. 3a; Fig. S3), or even a restricted dataset (Fig. 3b; Fig. S4) of the most closely adjacent clade of Asgard archaeal RadA (as seen in Fig. S3), we found the eukaryotic homologs always formed a well-supported monophyletic clade to the exclusion of the archaeal paralogues.

## Discussion

Our phylogenetic investigation shows that the duplication of an ancestral Asgard archaeal RadA gene, giving rise to Rad51 and DMC1, took place after the divergence of the eukaryotic lineage from the currently sampled Asgard archaea. If we assume that the Rad51 versus DMC1 duplication informs us about the evolution of meiosis and a sexual cycle, then the data suggest that this eukaryotic hallmark evolved from Asgard archaeal machinery after the divergence of the eukaryotic progenitor from the other Asgard archaeal lineages. Had we observed the duplication in the Asgard archaea, this would have implied meiotic capacity at an earlier stage, prior to the acquisition of the endosymbiont that gave rise to mitochondria.

From the start, it is worth clearly stating limitations to this study. Firstly, these conclusions are only true of the taxa that we sampled and could be overturned by the discovery of a new Asgard archaeal lineage, particularly one that branches more closely to eukaryotes than do the existing Heimdallarchaeota (Eme et al. 2023), if that organism encoded RadA paralogues that branch with the specific Rad51 and DMC1 clades. Such a lineage has not yet been discovered. Secondly, the assumed scenario of meiosis being tracked by this gene duplication presumes that meiotic function emerged as a eukaryotic feature, which entailed neofunctionalization following duplication. The findings here do not preclude the possibility that some of the meiosis-specific aspects of recombinase function evolved in the pre-duplicated form, as recently suggested elsewhere (Ferlez et al. 2025). Finally, we recognize that we are tracking only a single component of a complicated system, numbering dozens of proteins (Chen and Weir 2024). Understanding the emergence of each of these along the Asgard archaeal lineage, or indeed other prokaryotic origins, will fill in the complexity of the story of how meiotic sex evolved during eukaryogenesis. In particular, resolving exactly when eukaryotes acquired fusexins, most likely from the Euryarchaea, will be informative for understanding the evolution of cell fusion, which permitted whole genome syngamy (Moi et al. 2022). With these caveats in mind, our data nonetheless lend themselves to some important conclusions about how meiotic sex contributed to eukaryogenesis.

The most straightforward conclusion is that the duplication of RadA into general and meiosis-specific paralogues, heralding the evolution of sex, did not occur in the Asgard archaeal ancestor of the eukaryotes, but rather after the divergence of the eukaryotic line. This interpretation is consistent with theoretical considerations, which have shown that a large genome, beyond what is seen in bacteria and archaea, cannot be sustained by the genetic exchange facilitated by LGT. This key feature of the eukaryotes is only feasible with recombination between synapsed homologous chromosomes, as in meiosis (Colnaghi et al. 2020, 2022). This contrasts with features such as the cytoskeleton (Akıl and Robinson 2018; Wollweber et al. 2025) and a number of proteins involved in membrane trafficking and remodeling, notably a well-functioning ESCRT system (Souza et al. 2025) and an Asgard archaeal precursor to the Arf family GTPases (Vargová et al. 2025). These molecular and biochemical signatures have been confirmed in Asgard archaea, even though there is no indication to date that modern Asgard archaea possess extensive internal compartmentalization (Imachi et al. (2025) and Wollweber et al. (2025) but see MacLeod et al. (2025)). But the simplest assumption is that these proteins were integrated, establishing burgeoning endomembrane systems in early eukaryotes, fashioning their subsequent differentiation.

What do the results tell us about the evolution of sex in relation to eukaryogenesis? We are framing our discussion agnostic from specific eukaryogenic models (see Donoghue et al. (2023) for a review). Nonetheless, we do include the assumption that the mitochondrial endosymbiont provided energy that facilitated the emergence of various complex eukaryotic features, including the expansion of genome size, as seen in LECA (Lane and Martin 2010; Lane 2020). The endosymbiont's own genome shrank and massively moved into what became the nuclear genome, contributing to its expansion. At the same time, LGT alone has been modeled to be insufficient for the genome expansion necessary for the inferred state of multiple paralogues and extended genomic content in LECA (Lane 2011; Colnaghi et al. 2020, 2022). This modeling is consistent with the limited genome sizes of known Asgard archaea and the inferred gene content of ancestral Asgard archaea: there is presently no evidence for genome expansion toward eukaryotic-sized genomes before the acquisition of mitochondria or meiotic sex (Wu et al. 2022; Huang et al. 2025).

More work is needed to understand whether the pressure to increase genome size, engendered by the presence of the endosymbiont (Colnaghi et al. 2020; Lane 2020), promoted the evolution of meiotic sex or whether meiotic sex preceded and allowed a subsequent increase in genome size. Finally, cytoplasmic fusion and reciprocal recombination likely allowed genetic innovations arising in sub-populations of proto-eukaryotes to be brought together, ultimately giving rise to a relatively homogenous LECA (Záhonová et al. 2025) with a large number of traits not (yet) found in Asgard archaea or any other prokaryotes, including the nucleus, spliceosomal introns and exons, mitosis and meiosis, endomembrane systems, and more. Unlike LGT or cloning, reciprocal sex (with some form of cell fusion) can readily explain the accumulation of all these morphological traits in LECA (Lane 2011).

Overall, the data are consistent with some of the most significant genetic and biochemical capabilities underlying eukaryotic cellular configuration (e.g. cytoskeleton, membrane-trafficking proteins) evolving in the Asgard archaeal ancestor to eukaryotes. Powered by the acquisition of a symbiotic partner that eventually became the mitochondrion, sex enabled the evolution of a LECA with a large genome and unprecedented cellular complexity.

## Methods

### Dataset Assembly

The Comparative Set (TCS), comprising predicted amino acid (protein) sequences from 196 eukaryotic species, was obtained from EukProt v3 (https://evocellbio.com/eukprot/) (Leka and Wideman 2024). Non-Asgard archaeal sequences were retrieved from GenBank (https://www.ncbi.nlm.nih.gov/genbank/) and JGI (https://genome.jgi.doe.gov/portal/) deposit, while Asgard archaea sequences were collected from GenBank and the dataset published by (Eme et al. 2023). In total, archaeal sequences (Table S1) were collected from four major taxa: Euryarchaeota (70 species), DPANN (30 species), Asgard archaea (32 species), and TACK (24 species).

### Comparative Genomics Analyses

Hidden Markov Model (HMM) profiles were constructed and employed to identify homologous sequences using HMMer (Eddy 2009) which has greater sensitivity than BLAST (Altschul et al. 1997). Seed queries were used for initial sequence collection to build the respective HMMs, Rad51 (accession number: CAG38796) and DMC1 (accession number: CAG30372.1), and incorporated orthologs from eukaryotes in the TCS dataset which retrieved the relevant seed queries as a reciprocal best hit and greater than two orders of magnitude than the next best hit. Separate HMMs were built for Rad51 and DMC1. These profiles facilitated the detection and identification of Rad51 and DMC1 genes across the assembled eukaryotic and archaeal dataset. For comprehensive comparative genomic analyses, AMOEBAE (Analysis of MOlecular Evolution with BAtch Entry) was utilized (Barlow et al. 2023). This tool supports high-throughput homology searches and provides detailed analytical summaries, enabling efficient characterization of gene families across diverse taxa. It is freely available on GitHub (https://github.com/laelbarlow/amoebae).

### Phylogenetic Analyses

#### Eukaryotic Phylogenetic Tree

Eukaryotic sequences were aligned using MAFFT (Multiple Alignment using Fast Fourier Transform, v7.520; Katoh et al. 2019) with default settings (i.e. using FFT-NS-2 and BLOSUM62 matrix). The aligned sequences were refined with BMGE (Block Mapping and Gathering with Entropy) (Criscuolo and Gribaldo 2010) to remove poorly aligned regions and enhance the quality of the alignment. Both were accessed via the Galaxy Pasteur website (https://galaxy.pasteur.fr/) and used with default settings. This resulted in an alignment of 313 positions for 219 taxa which was used to construct a phylogenetic tree (Fig. S1) with IQ-TREE2 (multicore version 2.3.6, Aug 1 2024) (Minh et al. 2020) employing ModelFinder (Kalyaanamoorthy et al. 2017) with default (BIC) settings (automatically selected model: Q.insect+R7) and performing 1000 ultrafast bootstraps and 100 non-parametric bootstraps in parallel to assess branch support.

#### Archaeal Phylogenetic Tree

Asgard archaeal RadA sequences were selected for phylogenetic analyses, along with a smaller set of verified RadA homologs from diverse archaea other than Asgard archaea sourced from the UniProt database (https://www.uniprot.org/) (2025). To mitigate noise and phylogenetic artifacts, sequences contributing to long branches were removed from the dataset based on an initial phylogenetic tree constructed from the full set of RadA homologs (343 taxa, 300 positions, LG4X matrix, 1000 ultrafast bootstraps; Fig. S2), as were RadB sequences misidentified as RadA and duplicate sequences. The archaeal phylogenetic tree (Fig. 1) was then built by IQ-TREE2 with LG+C20 + G + F matrix based on the refined set of 85 RadA sequences (61 Asgard archaeal and 24 non-Asgard archaeal) aligned by MAFFT (default settings) and trimmed by Trimal (version 1.2rev59) (Capella-Gutiérrez et al. 2009) with the -gappyout option to the final length of 286 positions. Analyses using 1000 ultrafast bootstraps and 100 non-parametric bootstraps were run in parallel.

#### Combined Datasets

Three additional datasets were prepared using combinations of eukaryotic and archaeal homologs to assess the deeper relationships between RadA paralogs. For these analyses, the eukaryotic sampling was narrowed down to reduce computation time and sampling bias, using the initial tree (Fig. S2) as a reference, resulting in a dataset of 131 DMC1 and Rad51 homologs from 71 organisms. These sequences were then pooled with those from (i) all archaea (Fig. S3; tree of 216 taxa based on alignment of 307 positions), (ii) Asgard archaea (Fig. 2 tree of 192 taxa based on alignment of 306 positions), and (iii) a sub-sampling of Asgard archaea representing a presumed eukaryote-adjacent clade/paralogue of RadA (Fig. S4; tree of 142 taxa based on alignment of 329 positions). Alignment by MAFFT (default settings), trimming by Trimal (-gappyout option), and tree building by IQ-TREE2 with LG+C60+G+F matrix and parallel runs using 1000 ultrafast and 100 non-parametric bootstraps, respectively, were used for all three analyses.

## Supplementary Material

evaf240_Supplementary_Data

## Data Availability

Data and data description are available from https://figshare.com/articles/dataset/Sequence_datasets_alignments_and_phylogenetic_trees/29109308?file=54675668.
