# Supplementary material for: Eukaryotic Recombinases Duplicated After Divergence From Known Asgard Archaeal RadA: Implications for the Evolution of Sex During Eukaryogenesis
Source: Genome Biol Evol. 2025 Dec 23;17(12):evaf240. doi: 10.1093/gbe/evaf240 (PMC12722695; doi:10.1093/gbe/evaf240)
Supplement: evaf240_Supplementary_Data [file evaf240_supplementary_data.zip › Matsuo.SupplementaryFigures.R1.pdf]

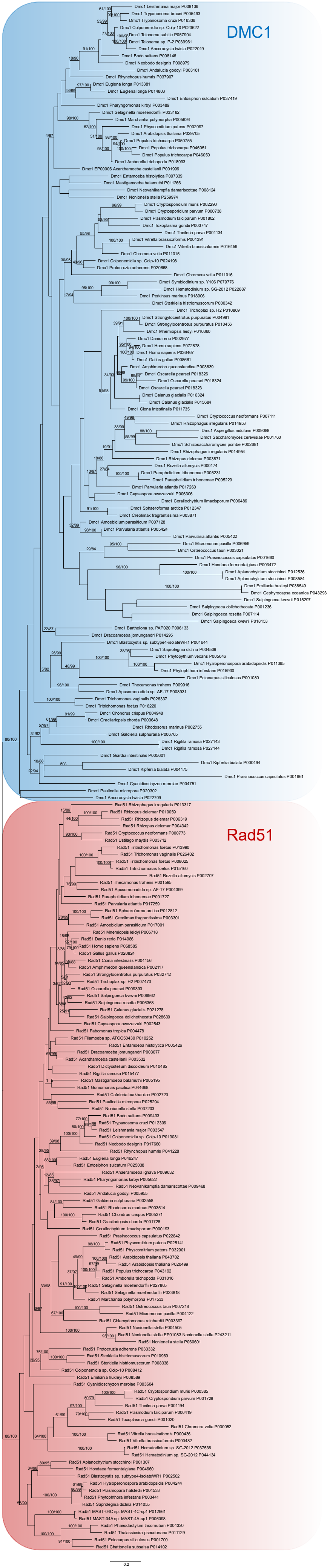

Figure S1: Rad51 and DMC1 are robustly resolved into separate clades encompassing the breadth of eukaryotes. This phylogenetic analysis sampled a wide range of eukaryotic taxa and resolved paralogues into clades of Rad51 (red shading) and DMC1 (blue shading). Support values for 100 NP boostraps and % of 1000 UF boostraps for IQ-TREE analyses are shown at each node supported by greater than 50 NP or 80 UF.

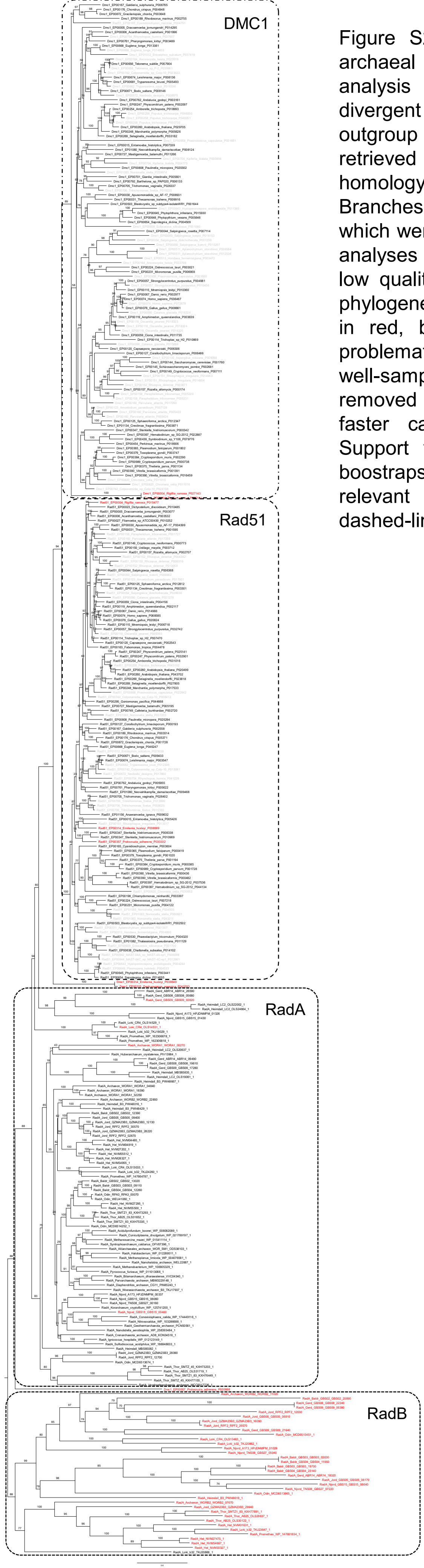

Figure S2: Analysis of all sampled archaeal Rad protein sequences. This analysis identified identical or highly divergent sequences, as well as RadB outgroup sequences erroneously retrieved by the highly sensitive homology searching approach. Branches representing sequences which were removed from subsequent analyses because of misidentification, low quality and/or potential to cause phylogenetic artifacts are highlighted in red, branches representing non-problematic sequences for otherwise well-sampled groups which were removed for dataset conciseness and faster calculation are greyed out. Support values for % of 1000 UF boostraps from IQ-TREE analysis and relevant clades are denoted by dashed-line boxes.

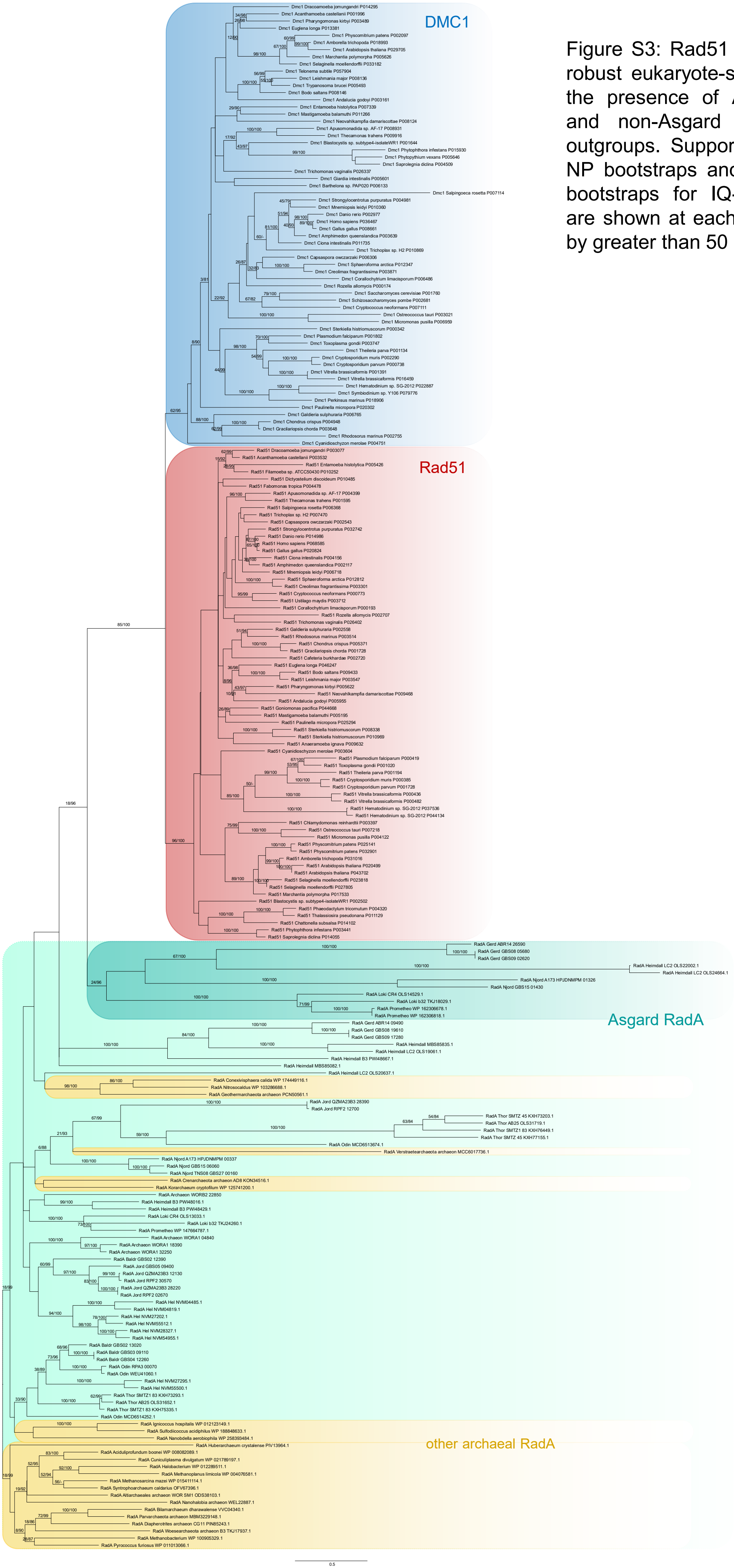

Figure S3: Rad51 and DMC1 form robust eukaryote-specific clades in the presence of Asgard archaeal and non-Asgard archaeal RadA outgroups. Support values for 100 NP bootstraps and % of 1000 UF bootstraps for IQ-TREE analyses are shown at each node supported by greater than 50 NP or 80 UF.

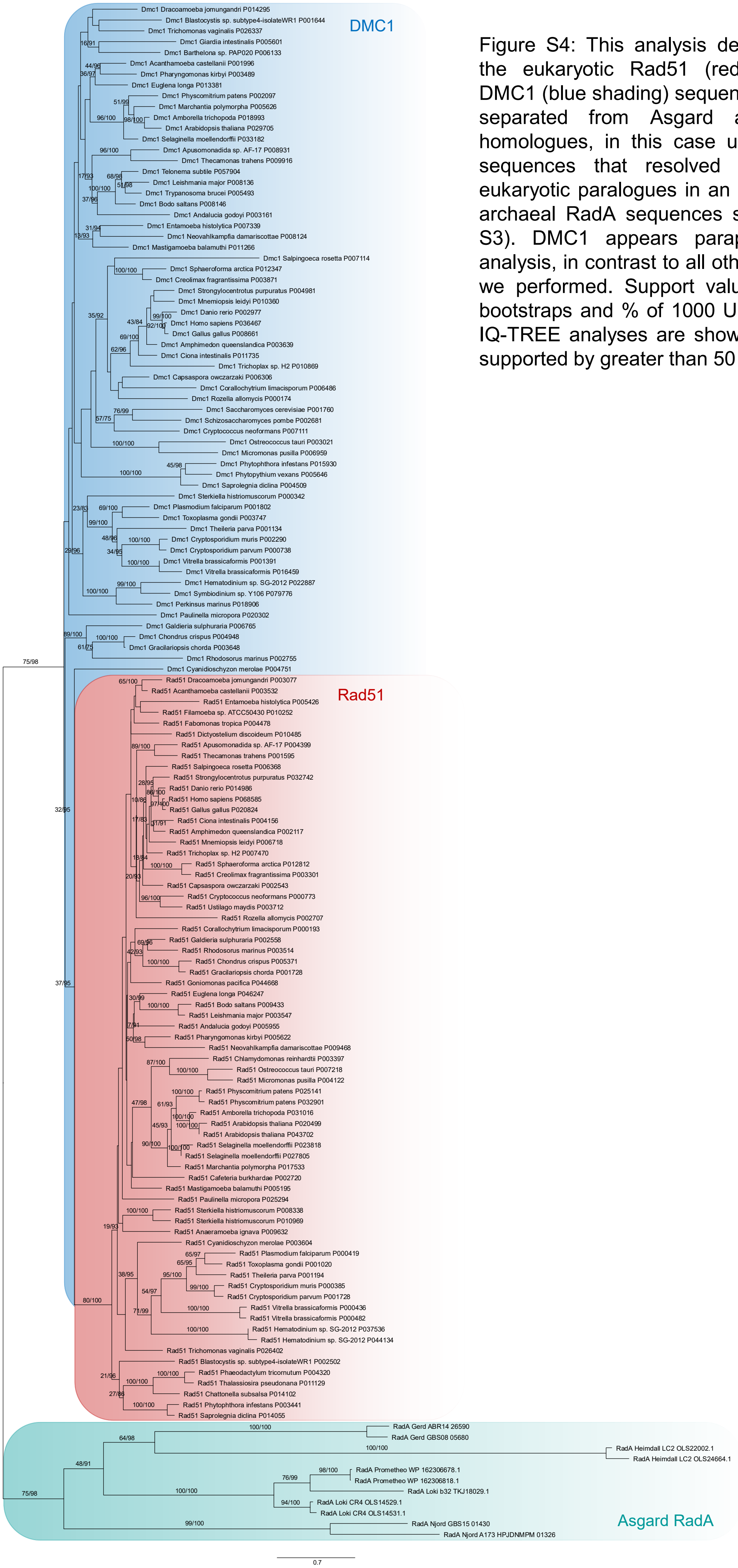

Figure S4: This analysis demonstrates that the eukaryotic Rad51 (red shading) and DMC1 (blue shading) sequences are robustly separated from Asgard archaeal RadA homologues, in this case using only those sequences that resolved closest to the eukaryotic paralogues in an analysis with all archaeal RadA sequences sampled (Figure S3). DMC1 appears paraphyletic in this analysis, in contrast to all other analyses that we performed. Support values for 100 NP bootstraps and % of 1000 UF bootstraps for IQ-TREE analyses are shown at each node supported by greater than 50 NP or 80 UF.
